# Supplementary material for: Reconstructing skeletal homeostasis through allogeneic hematopoietic stem cell transplantation in myelofibrosis
Source: Nat Commun. 2025 Jan 20;16:741. doi: 10.1038/s41467-025-55915-w (PMC11747566; doi:10.1038/s41467-025-55915-w)
Supplement: Supplementary file 1 — Supplementary information [file 41467_2025_55915_MOESM1_ESM.pdf]

Supplementary information

Reconstructing skeletal homeostasis through allogeneic hematopoietic stem cell transplantation in myelofibrosis

Mathias Schäfersküpper*, Alexander Simon*, Timur A. Yorgan, Felix N. von Brackel, Maximilian M. Delsmann, Anke Baranowsky, Nico Gagelmann, Francis Ayuk, Thorsten Schinke, Michael Amling, Nicolaus Kröger#, and Tim Rolvien#

*These authors contributed equally: Mathias Schäfersküpper, Alexander Simon

#These authors jointly supervised this work: Nicolaus Kröger, Tim Rolvien

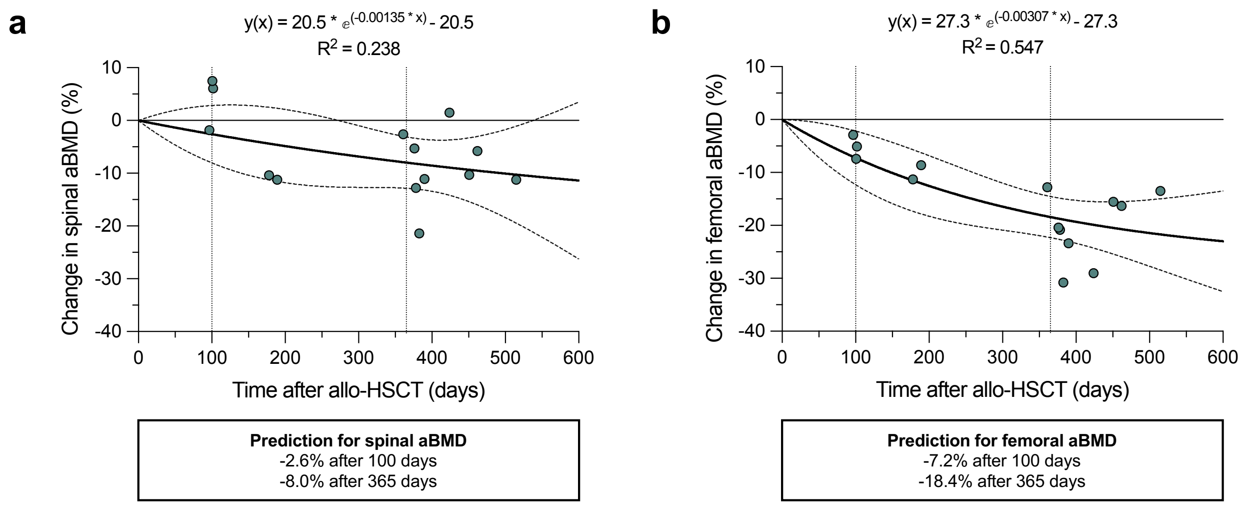

Supplementary Figure 1. Non-linear regression analyses of longitudinal changes in spinal and femoral areal bone mineral density (aBMD) assessed by dual-energy X-ray absorptiometry (DXA) in myelofibrosis after allogeneic hematopoietic stem cell transplantation (allo-HSCT). a Percentage changes in spinal aBMD at different time intervals following allo-HSCT and non-linear regression analysis (n = 14). **b** Percentage changes in femoral aBMD at different time intervals following allo-HSCT and non-linear regression analysis (n = 14). All data points and non-linear regression analyses (solid lines) including 95% confidence intervals (dotted lines) are displayed. The predictive equations for the percentage change in aBMD after allo-HSCT with the coefficient of determination R^2 are shown above the graphs. Calculated percentage changes in aBMD at 100 days and 365 days after allo-HSCT for both sites are denoted below the graphs. Source data are provided as a Source Data file.

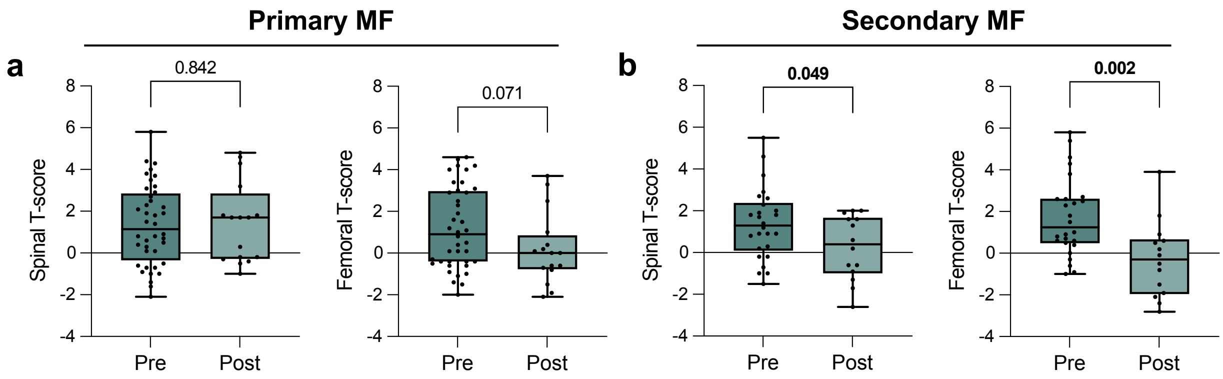

Supplementary Figure 2. T-score assessed by dual-energy X-ray absorptiometry (DXA) in primary and secondary myelofibrosis (MF) before and after allogeneic hematopoietic stem cell transplantation (allo-HSCT). a Spinal and femoral T-score before (Pre, n = 40) and after allo-HSCT (Post, n = 16) in patients with primary MF. **b** Spinal and femoral T-score before (Pre, n = 26) and after allo-HSCT (Post, n = 14) in patients with secondary MF. The median (center line; 50th percentile) and interquartile range (box edges; 25th to 75th percentile) with whiskers extending to the minimum and maximum values are depicted. Each data point is shown. Differences between two groups were calculated using unpaired two-tailed *t* test (a spinal T-score, b) was used for normally distributed data and the Mann–Whitney *U* test (a femoral T-score) was used for non-parametric data. Exact *p*-values of the comparisons are displayed above the brackets and numbers in bold indicate statistical significance ($p < 0.05$). Source data are provided as a Source Data file.

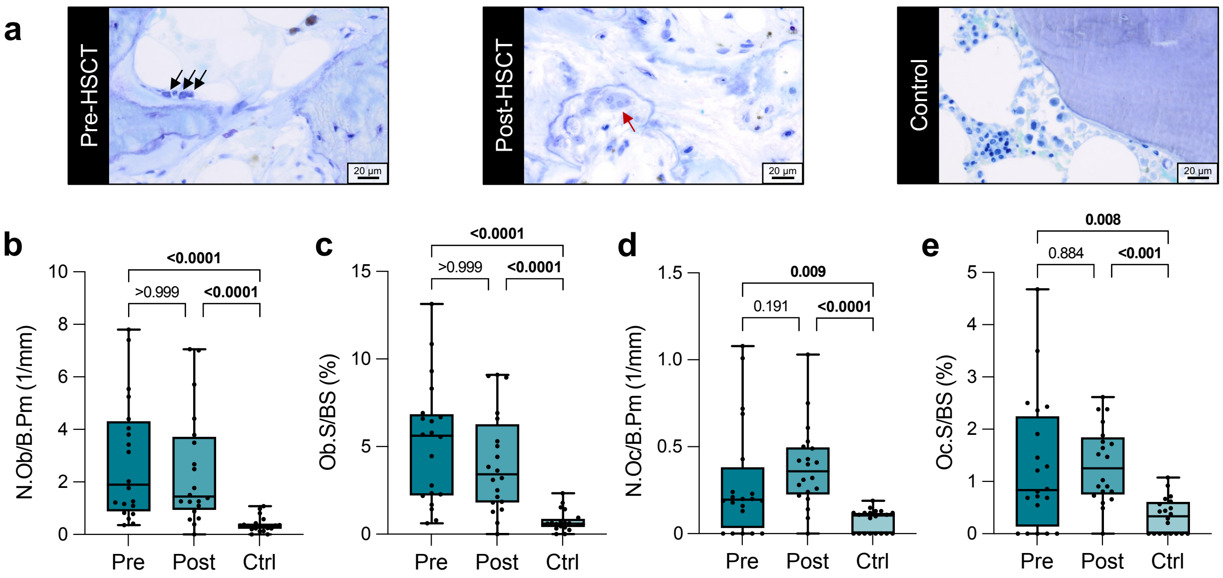

Supplementary Figure 3. Persistent histomorphometric high bone turnover in myelofibrosis after allo-HSCT. a Representative histological images of toluidine blue-stained sections in myelofibrosis patients before allo-HSCT (Pre-HSCT) and after allo-HSCT (Post-HSCT), along with the control group (Control). The black arrows indicate osteoblasts and the red arrow an osteoclast. **b** Number of osteoblasts per bone perimeter (N.Ob/B.Pm) of myelofibrosis patients before allo-HSCT (Pre, n = 20) and after allo-HSCT (Post, n = 20), along with the control group (Ctrl, n = 20). **c** Osteoblast surface to bone surface (Ob.S/BS) of the three groups (Pre, n = 20; Post, n = 20; Ctrl, n = 20). **d** Number of osteoclasts per bone perimeter (N.Oc/B.Pm) of the three groups (Pre, n = 20; Post, n = 20; Ctrl, n = 20). **e** Osteoclast surface to bone surface (Oc.S/BS) of the three groups (Pre, n = 20; Post, n = 20; Ctrl, n = 20). The median (center line; 50th percentile) and interquartile range (box edges; 25th to 75th percentile) with whiskers extending to the minimum and maximum values are depicted. Each data point is shown. Differences between the three groups were calculated using Kruskal-Wallis *H* test with Dunn's test (b-e). Exact *p*-values of the comparisons are displayed above the brackets and numbers in bold indicate statistical significance ($p < 0.05$). Source data are provided as a Source Data file.

Supplementary Table 1. Clinical characteristics of the study cohort prior to allo-HSCT.

Parameter	Total n = 66	Women n = 34	Men n = 32	*p*	*ω*
**Diagnosis**					
PMF	40 (60.6%)	17 (50.0%)	23 (71.9%)		
Post-ET MF	16 (24.2%)	12 (35.3%)	4 (12.5%)	0.089	0.27
Post-PV MF	10 (15.2%)	5 (14.7%)	5 (15.6%)		
**MF grade**					
MF-3	48 (72.7%)	25 (73.5%)	23 (71.9%)		
MF-2	13 (19.7%)	8 (23.5%)	5 (15.6%)	0.352	0.20
MF-1	5 (7.6%)	1 (2.9%)	4 (12.5%)		
MF-0	0 (0.0%)	0 (0.0%)	0 (0.0%)		
**DIPPS/MYSEC-PM**					
High risk	14 (21.2%)	7 (20.6%)	7 (21.9%)		
Intermediate-2 risk	40 (60.6%)	18 (52.9%)	22 (68.8%)	0.298	0.23
Intermediate-1 risk	11 (16.7%)	8 (23.5%)	3 (9.4%)		
Low risk	1 (1.5%)	1 (2.9%)	0 (0.0%)		
**Conditioning regimen**					
RIC	58 (87.9%)	31 (91.2%)	27 (84.4%)	0.469	0.10
MAC	8 (12.1%)	3 (8.8%)	5 (15.6%)		
**Donor type**					
Matched donor	56 (84.8%)	27 (79.4%)	29 (90.6%)	0.306	0.16
Mismatched donor	10 (15.2%)	7 (20.6%)	3 (9.4%)		

Abbreviations: Allo-HSCT, Allogeneic hematopoietic stem cell transplantation. DIPPS, Dynamic international prognostic scoring system for myelofibrosis. MAC, Myeloablative conditioning. MF grade, Bone marrow fibrosis grade. MYSEC-PM, Myelofibrosis secondary to PV and ET prognostic model. PMF, Primary myelofibrosis. Post-ET MF, Post-essential thrombocythemia myelofibrosis. Post-PV MF, Post-polycythemia vera myelofibrosis. RIC, Reduced-intensity conditioning.

Absolute values are displayed with corresponding percentages. Differences in the frequency distribution between women and men were tested using two-tailed Fisher's exact test, Exact *p*-values with corresponding effect sizes *ω* (0.1 ≙ small, 0.3 ≙ medium, 0.5 ≙ large effect size) are presented.

Supplementary Table 2. Demographic and densitometric characteristics of the study cohort prior to allo-HSCT and the propensity score matched healthy control group.

	**MF** (n = 66)				**Control** (n = 66)					
Parameter	Mean	SD	Min	Max	Mean	SD	Min	Max	*p*	*ω* | d
**Demographics**										
Sex (female / male)	34/32 (51.5%/48.5%)				34/32 (51.5%/48.5%)				>0.999	0.00
Age (years)	60.3	8.7	42	75	60.5	8.8	42	75	0.909	0.02
Weight (kg)	77.9	16.4	47.6	135.0	77.3	15.1	48.5	111.0	0.825	0.04
Height (m)	1.73	0.10	1.50	1.95	1.73	0.10	1.51	1.94	0.908	0.00
BMI (kg/m^2)	25.9	4.4	17.7	37.8	25.7	4.1	18.8	38.6	0.907	0.02
**DXA**										
Spinal aBMD (g/cm^2)	1.367	0.226	0.969	1.876	1.195	0.149	0.932	1.666	**<0.0001**	0.86
Spinal T-score	1.4	1.8	-2.1	5.8	0.0	1.2	-2.1	3.7	**<0.0001**	0.88
Spinal Z-score	1.9	1.8	-1.6	6.3	0.5	1.0	-0.8	3.4	**<0.0001**	0.91
Femoral aBMD (g/cm^2)	1.220	0.249	0.819	1.693	0.969	0.120	0.756	1.251	**<0.0001**	1.23
Femoral T-score	1.4	1.9	-2.0	5.8	-0.6	0.8	-2.0	1.4	**<0.0001**	1.35
Femoral Z-score	2.1	1.8	-1.3	6.0	0.0	0.6	-1.0	1.5	**<0.0001**	1.57
Normal BMD	47 of 66 (71.2%)				25 of 66 (37.9%)					
Osteopenia (< -1.0)	19 of 66 (28.8%)				39 of 66 (59.1%)				**<0.001**	0.34
Osteoporosis (≤ -2.5)	0 of 66 (0.0%)				2 of 66 (3.0%)					

Abbreviations: Allo-HSCT, Allogeneic hematopoietic stem cell transplantation. aBMD, Areal bone mineral density. BMI, Body mass index. DXA, Dual-energy X-ray absorptiometry. MF, Myelofibrosis. SD, Standard deviation.

Patients were classified into normal BMD, osteopenia, and osteoporosis based on the lowest T-score of any site. Means ± standard deviations with corresponding minimal and maximal values are displayed. Differences between groups were tested using two-tailed unpaired *t* test or Mann-Whitney *U* test. *p*-values with corresponding effect sizes Cohen's d (0.2 ≙ small, 0.5 ≙ medium, 0.8 ≙ large effect size) are presented. For sex and BMD groups absolute values with corresponding percentages are displayed. Differences in the frequency distribution between MF patients and controls were tested using two-tailed Fisher's exact test. Exact *p*-values with corresponding effect sizes *ω* (0.1 ≙ small, 0.3 ≙ medium, 0.5 ≙ large effect size) are presented. Numbers in bold indicate statistical significance ($p < 0.05$). Source data are provided as a Source Data file.

Supplementary Table 3. Sex-specific demographic and densitometric characteristics of the study cohort prior to allo-HSCT and the propensity score matched healthy control group.

	Women						Men					
	MF (n = 34)		Control (n = 34)				MF (n = 32)		Control (n = 32)			
Parameter	Mean	SD	Mean	SD	*p*	d | *ω*	Mean	SD	Mean	SD	*p*	d | *ω*
**Demographics**												
Age (years)	59.5	8.6	59.7	8.5	0.932	0.02	61.2	8.8	61.4	9.2	0.957	0.01
Weight (kg)	69.4	12.3	69.0	11.8	0.908	0.03	87.0	15.3	86.1	13.3	0.830	0.05
Height (m)	1.67	0.08	1.67	0.07	0.662	0.00	1.80	0.07	1.79	0.08	0.800	0.13
BMI (kg/m^2)	25.0	4.3	24.7	3.9	0.745	0.07	26.9	4.3	26.9	4.1	0.950	0.00
**DXA**												
Spinal aBMD (g/cm^2)	1.312	0.210	1.137	0.133	**<0.001**	0.96	1.425	0.232	1.258	0.141	**0.002**	0.83
Spinal T-score	1.1	1.7	-0.4	1.1	**<0.001**	0.93	1.7	1.9	0.3	1.2	**0.002**	0.85
Spinal Z-score	2.0	1.7	0.6	0.9	**<0.001**	1.04	1.8	1.9	0.5	1.1	**0.003**	0.81
Femoral aBMD (g/cm^2)	1.157	0.229	0.900	0.096	**<0.0001**	1.73	1.286	0.256	1.043	0.098	**<0.001**	1.03
Femoral T-score	1.3	1.9	-0.8	0.8	**<0.0001**	1.72	1.5	2.0	-0.4	0.8	**<0.001**	1.05
Femoral Z-score	2.1	1.8	-0.1	0.6	**<0.0001**	1.64	2.0	1.9	0.2	0.6	**<0.001**	1.09
Normal BMD	24 of 34 (70.6%)		8 of 34 (23.5%)				23 of 32 (71.9%)		17 of 32 (53.1%)			
Osteopenia (< -1.0)	10 of 34 (29.4%)		24 of 34 (70.6%)		**<0.001**	0.48	9 of 32 (28.1%)		15 of 32 (46.9%)		0.196	0.19
Osteoporosis (≤ -2.5)	0 of 34 (0.0%)		2 of 34 (5.9%)				0 of 32 (0.0%)		0 of 32 (0.0%)			

Abbreviations: Allo-HSCT, Allogeneic hematopoietic stem cell transplantation. aBMD, Areal bone mineral density. BMI, Body mass index. DXA, Dual-energy X-ray absorptiometry. MF, Myelofibrosis. SD, Standard deviation.

Patients were classified into normal BMD, osteopenia, and osteoporosis based on lowest T-score of any site. Means ± standard deviations are displayed. Sex-specific differences between MF patients prior to allo-HSCT and controls were tested using two-tailed unpaired *t* test or Mann-Whitney *U* test. *p*-values with corresponding effect sizes Cohen's d (0.2 ≙ small, 0.5 ≙ medium, 0.8 ≙ large effect size) are presented. For BMD groups absolute values with corresponding percentages are displayed. Sex-specific differences in the frequency distribution between MF patients and controls were tested using two-tailed Fisher's exact test. Exact *p*-values with corresponding effect sizes *ω* (0.1 ≙ small, 0.3 ≙ medium, 0.5 ≙ large effect size) are presented. Numbers in bold indicate statistical significance ($p < 0.05$). Source data are provided as a Source Data file.

Supplementary Table 4. Densitometric, microarchitectural, and geometric bone parameters assessed by HR-pQCT of the study cohort prior to allo-HSCT and the propensity score matched control group.

	Radius								Tibia							
	**MF** (n = 57)			**Control** (n = 57)					**MF** (n = 57)			**Control** (n = 57)				
Parameter	Mean	SD	% mean[1]	Mean	SD	% mean[1]	*p*	d	Mean	SD	% mean[1]	Mean	SD	% mean[1]	*p*	d
**HR-pQCT**																
Tt.vBMD (mg HA/cm³)	308.3	68.1	96.7	305.1	60.9	95.9	0.792	0.05	282.3	66.3	101.9	279.6	43.9	101.1	0.793	0.05
Tt.Ar (mm²)	340.3	77.2	111.0	334.5	77.6	109.2	0.421	0.15	822.8	144.3	103.3	832.8	176.7	104.4	0.937	0.01
Tb.vBMD (mg HA/cm³)	162.7	50.9	108.2	164.5	42.0	109.8	0.837	0.04	175.7	53.5	107.0	169.5	36.7	103.3	0.472	0.14
BV/TV (%)	13.6	4.2	108.2	13.7	3.5	109.9	0.836	0.03	14.6	4.5	106.9	14.1	3.1	103.3	0.471	0.13
Tb.N (mm⁻¹)	1.99	0.35	104.0	2.04	0.35	107.1	0.292	0.20	2.03	0.43	103.1	1.97	0.42	100.0	0.441	0.14
Tb.Th (mm)	0.067	0.013	103.3	0.067	0.011	102.7	0.894	0.03	0.072	0.015	103.1	0.073	0.011	104.2	0.772	0.08
Tb.Sp (mm)	0.455	0.118	99.3	0.441	0.114	96.1	0.371	0.17	0.442	0.109	99.4	0.466	0.158	104.4	0.687	0.08
Tb.Ar (mm²)	264.0	70.2	113.7	262.7	72.2	112.9	0.700	0.07	683.9	138.3	103.3	690.8	165.4	104.3	0.935	0.02
Ct.vBMD (mg HA/cm³)	803.5	77.9	91.2	783.4	97.0	88.9	0.245	0.22	790.5	73.4	94.2	795.0	48.6	94.8	0.702	0.07
Ct.Th (mm)	0.73	0.23	75.7	0.71	0.19	73.6	0.559	0.09	1.04	0.32	86.2	1.06	0.23	89.0	0.628	0.07
Ct.Pm (mm)	79.9	10.7	-	79.6	11.1	-	0.489	0.13	114.2	10.5	-	114.2	12.6	-	0.996	0.00
Ct.Ar (mm²)	58.5	20.2	88.2	56.0	15.4	85.5	0.459	0.14	118.1	38.1	92.8	121.0	28.3	96.2	0.650	0.09

Abbreviations: Allo-HSCT, Allogeneic hematopoietic stem cell transplantation. BV/TV, Bone volume to tissue volume. Ct.Ar, Cortical area. Ct.vBMD, Cortical volumetric bone mineral density. Ct.Pm, Cortical perimeter. Ct.Th, Cortical thickness. HR-pQCT, High-resolution peripheral quantitative computed tomography. MF, Myelofibrosis. SD, Standard deviation. Tb.Ar, Trabecular area. Tb.vBMD, Trabecular volumetric bone mineral density. Tb.N, Trabecular number. Tb.Sp, Trabecular separation. Tb.Th, Trabecular thickness. Tt.Ar, Total area. Tt.vBMD, Total volumetric bone mineral density.

Means ± standard deviations with mean percentages of the median of age- and sex-specific reference values[1] are displayed. Differences between MF patients prior to allo-HSCT and controls were tested using two-tailed unpaired *t* test or Mann-Whitney *U* test. Exact *p*-values with corresponding effect sizes Cohen's d (0.2 ≙ small, 0.5 ≙ medium, 0.8 ≙ large effect size) are presented. Source data are provided as a Source Data file.

Supplementary Table 5. Densitometric, microarchitectural, and geometric bone parameters assessed by HR-pQCT of the female study cohort prior to allo-HSCT and the propensity score matched female control group.

**Women**	**Radius**								**Tibia**							
	**MF** (n = 29)			**Control** (n = 29)					**MF** (n = 29)			**Control** (n = 29)				
Parameter	Mean	SD	% mean[1]	Mean	SD	% mean[1]	*p*	d	Mean	SD	% mean[1]	Mean	SD	% mean[1]	*p*	d
**HR-pQCT**																
Tt.vBMD (mg HA/cm^3)	285.2	65.5	93.3	291.4	60.9	95.2	0.711	0.10	253.8	58.5	100.3	257.6	41.0	101.7	0.773	0.08
Tt.Ar (mm^2)	287.1	52.9	115.4	280.1	36.9	112.8	0.559	0.15	753.1	129.2	108.8	723.8	106.3	104.7	0.350	0.25
Tb.vBMD (mg HA/cm^3)	137.4	40.8	106.7	142.8	38.3	110.6	0.604	0.14	153.9	50.2	104.4	145.1	29.3	98.8	0.791	0.07
BV/TV (%)	11.4	3.4	106.8	11.9	3.2	110.8	0.599	0.15	12.8	4.2	104.5	12.1	2.4	99.0	0.797	0.07
Tb.N (mm^{-1})	1.82	0.31	102.2	1.86	0.34	104.1	0.661	0.12	1.88	0.33	100.0	1.76	0.40	93.7	0.504	0.17
Tb.Th (mm)	0.062	0.011	103.6	0.064	0.011	106.5	0.580	0.15	0.068	0.016	103.3	0.070	0.012	107.6	0.259	0.30
Tb.Sp (mm)	0.505	0.126	102.1	0.495	0.129	99.8	0.419	0.21	0.477	0.090	101.2	0.536	0.186	113.7	0.474	0.19
Tb.Ar (mm^2)	226.7	55.1	123.1	218.4	38.2	119.1	0.505	0.18	637.6	133.4	109.7	601.1	103.5	103.6	0.248	0.31
Ct.vBMD (mg HA/cm^3)	795.3	83.4	89.4	783.0	114.6	88.1	0.785	0.07	774.5	70.6	93.4	793.3	51.1	95.7	0.250	0.31
Ct.Th (mm)	0.65	0.20	69.8	0.68	0.17	73.0	0.571	0.16	0.88	0.24	81.3	0.98	0.20	90.5	0.102	0.45
Ct.Pm (mm)	72.3	7.1	-	71.6	4.7	-	0.660	0.12	108.7	9.5	-	106.0	8.0	-	0.248	0.31
Ct.Ar (mm^2)	46.4	12.1	84.0	48.4	11.0	87.7	0.508	0.17	95.1	23.5	93.3	103.7	21.2	102.0	0.148	0.38

Abbreviations: Allo-HSCT, Allogeneic hematopoietic stem cell transplantation. BV/TV, Bone volume to tissue volume. Ct.Ar, Cortical area. Ct.vBMD, Cortical volumetric bone mineral density. Ct.Pm, Cortical perimeter. Ct.Th, Cortical thickness. HR-pQCT, High-resolution peripheral quantitative computed tomography. MF, Myelofibrosis. SD, Standard deviation. Tb.Ar, Trabecular area. Tb.vBMD, Trabecular volumetric bone mineral density. Tb.N, Trabecular number. Tb.Sp, Trabecular separation. Tb.Th, Trabecular thickness. Tt.Ar, Total area. Tt.vBMD, Total volumetric bone mineral density.

Means ± standard deviations with mean percentages of the median of age- and sex-specific reference values[1] are displayed. Differences between female MF patients prior to allo-HSCT and female controls were tested using two-tailed unpaired *t* test or Mann-Whitney *U* test. Exact *p*-values with corresponding effect sizes Cohen's d (0.2 ≙ small, 0.5 ≙ medium, 0.8 ≙ large effect size) are presented. Source data are provided as a Source Data file.

Supplementary Table 6. Densitometric, microarchitectural, and geometric bone parameters assessed by HR-pQCT of the male study cohort prior to allo-HSCT and the propensity score matched male control group.

**Men**	**Radius**								**Tibia**							
	**MF** (n = 28)			**Control** (n = 28)					**MF** (n = 28)			**Control** (n = 28)				
Parameter	Mean	SD	% mean[1]	Mean	SD	% mean[1]	*p*	d	Mean	SD	% mean[1]	Mean	SD	% mean[1]	*p*	d
**HR-pQCT**																
Tt.vBMD (mg HA/cm^3)	332.1	63.3	100.3	319.2	58.7	96.6	0.432	0.21	311.9	61.5	103.5	302.3	34.8	100.5	0.446	0.20
Tt.Ar (mm^2)	395.4	57.1	106.5	390.9	67.8	105.6	0.786	0.07	895.0	123.5	97.6	945.8	164.3	104.1	0.196	0.35
Tb.vBMD (mg HA/cm^3)	189.0	47.3	109.7	187.1	33.2	108.9	0.859	0.05	198.3	47.8	109.6	194.8	24.4	107.9	0.731	0.09
BV/TV (%)	15.8	3.9	109.8	15.6	2.8	108.9	0.854	0.06	16.5	4.0	109.4	16.2	2.0	107.7	0.731	0.09
Tb.N (mm^{-1})	2.15	0.31	105.9	2.23	0.25	110.1	0.300	0.28	2.18	0.47	106.3	2.18	0.33	106.6	0.989	0.00
Tb.Th (mm)	0.073	0.013	103.0	0.070	0.009	98.9	0.646	0.12	0.076	0.013	102.9	0.075	0.009	100.7	0.649	0.09
Tb.Sp (mm)	0.402	0.082	96.4	0.384	0.054	92.2	0.617	0.13	0.406	0.118	97.6	0.394	0.072	94.8	0.712	0.10
Tb.Ar (mm^2)	302.6	63.5	104.0	308.7	70.6	106.5	0.733	0.09	731.8	128.7	96.6	783.7	167.5	105.0	0.199	0.35
Ct.vBMD (mg HA/cm^3)	812.0	72.3	93.0	783.9	76.9	89.7	0.142	0.40	807.1	73.7	95.1	796.7	46.7	93.8	0.255	0.31
Ct.Th (mm)	0.82	0.24	81.8	0.74	0.21	74.3	0.216	0.35	1.19	0.31	91.2	1.14	0.23	87.4	0.500	0.18
Ct.Pm (mm)	87.8	7.6	-	87.9	9.5	-	0.958	0.01	119.8	8.5	-	122.6	10.9	-	0.288	0.29
Ct.Ar (mm^2)	71.1	19.2	92.6	64.0	15.4	83.3	0.128	0.41	141.9	35.8	92.2	138.8	23.4	90.2	0.703	0.10

Abbreviations: Allo-HSCT, Allogeneic hematopoietic stem cell transplantation. BV/TV, Bone volume to tissue volume. Ct.Ar, Cortical area. Ct.vBMD, Cortical volumetric bone mineral density. Ct.Pm, Cortical perimeter. Ct.Th, Cortical thickness. HR-pQCT, High-resolution peripheral quantitative computed tomography. MF, Myelofibrosis. SD, Standard deviation. Tb.Ar, Trabecular area. Tb.vBMD, Trabecular volumetric bone mineral density. Tb.N, Trabecular number. Tb.Sp, Trabecular separation. Tb.Th, Trabecular thickness. Tt.Ar, Total area. Tt.vBMD, Total volumetric bone mineral density.

Means ± standard deviations with mean percentages of the median of age- and sex-specific reference values[1] are displayed. Differences between male MF patients prior to allo-HSCT and male controls were tested using two-tailed unpaired *t* test or Mann-Whitney *U* test. Exact *p*-values with corresponding effect sizes Cohen's d (0.2 ≙ small, 0.5 ≙ medium, 0.8 ≙ large effect size) are presented. Source data are provided as a Source Data file.

Supplementary Table 7. Demographic, bone histomorphometric and mineralization characteristics of the propensity score matched groups of myelofibrosis patients prior to allo-HSCT, subsequent to allo-HSCT, and the healthy control group.

	**Pre-HSCT** (n = 20)		**Post-HSCT** (n = 20)		**Control** (n = 20)			
Parameter	Mean	SD	Mean	SD	Mean	SD	*p*	η^2 | ω
**Demographics**								
Sex (female/male)	7/13 (35%/65%)		6/14 (30%/70%)		7/13 (35%/65%)		> 0.999	0.05
Age (years)	58.1	9.6	60.6	10.4	58.0	11.6	0.670	0.01
BMI (kg/m^2)	25.8	4.9	24.4	2.8	25.4	3.9	0.538	0.02
25-OH-D (µg/l)	19.9	11.8	26.0	12.2	19.7	11.2	0.191	0.02
**Bone Marrow**								
Fb.Ar/Ma.Ar (%)	71.0	25.5	5.5	5.9	0.8	1.4	**< 0.0001**	0.79
Ad.Ar/Ma.Ar (%)	20.5	21.2	55.3	23.2	46.3	14.3	**< 0.0001**	0.33
**Structure**								
BV/TV (%)	35.8	14.9	16.5	8.0	19.9	4.6	**< 0.0001**	0.42
Md.V/TV (%)	34.1	14.7	16.2	8.0	19.7	4.5	**< 0.0001**	0.39
Tb.N (1/mm)	2.25	1.18	1.31	0.58	1.24	0.26	**0.004**	0.16
Tb.Th (µm)	178.2	70.2	130.5	54.5	171.4	43.9	**0.006**	0.14
Tb.Sp (µm)	401.1	288.6	725.2	277.0	673.8	172.0	**< 0.001**	0.21
**Osteoid**								
OV/BV (%)	4.9	6.5	1.2	1.3	1.2	1.0	0.155	0.03
Osteomalacia	10 of 20 (50%)		2 of 20 (10%)		5 of 20 (25%)		**0.023**	0.37
OS/BS (%)	25.2	25.4	9.1	7.2	8.8	6.2	0.065	0.06
O.Th (µm)	11.80	4.95	9.27	5.73	11.30	4.49	0.257	0.05
**Cells**								
N.Ob/B.Pm (1/mm)	2.82	2.32	2.43	2.15	0.37	0.29	**< 0.0001**	0.49
Ob.S/BS (%)	5.19	3.48	4.04	2.83	0.75	0.60	**< 0.0001**	0.48
N.Oc/B.Pm (1/mm)	0.29	0.33	0.38	0.24	0.07	0.07	**< 0.0001**	0.38
Oc.S/BS (%)	1.28	1.28	1.32	0.73	0.34	0.35	**< 0.001**	0.28
**Mineralization**								
CaMean (wt%)	23.06	1.63	23.49	1.23	24.73	0.86	**< 0.0001**	0.32
CaWidth (wt%)	3.58	0.42	3.42	0.34	2.89	0.24	**< 0.0001**	0.49
CaLow (%)	16.08	13.64	12.34	9.00	4.97	3.93	**< 0.0001**	0.40
CaHigh (%)	3.19	2.41	3.38	1.97	4.98	3.14	0.065	0.06
**Osteocyte Lacunae**								
N.Ot.Lc/B.Ar (1/mm^2)	249.0	38.0	219.3	46.2	162.9	31.6	**< 0.0001**	0.47
Ot.Lc.Ar (µm^2)	32.03	2.27	31.22	3.86	29.03	2.21	**0.005**	0.17

Abbreviations: Ad.Ar/Ma.Ar, Adipose area to marrow area. Allo-HSCT, Allogeneic hematopoietic stem cell transplantation. BMI, Body mass index. BV/TV, Bone volume to tissue volume. CaHigh, Fraction of highly mineralized matrix. CaLow, Fraction of lowly mineralized matrix. CaMean, Mean calcium content. CaWidth, Mineralization heterogeneity. Fb.Ar/Ma.Ar, Fibrosis area to marrow area. Md.V/TV, Mineralized volume to tissue volume. N.Ob/B.Pm, Number of osteoblasts per bone perimeter. N.Oc/B.Pm, Number of osteoclasts per bone perimeter. N.Ot.Lc/B.Ar, Number of osteocyte lacunae per bone area. O.Th, Osteoid thickness. Ob.S/BS, Osteoblast surface to bone surface. Oc.S/BS, Osteoclast surface to bone surface. OS/BS, Osteoid surface to bone surface. Ot.Lc.Ar, Osteocyte lacunar area. OV/BV, Osteoid volume to bone volume. SD, Standard deviation. Tb.N, Trabecular number. Tb.Sp, Trabecular separation. Tb.Th, Trabecular thickness. 25-OH-D, 25-hydroxyvitamin D.

Osteomalacia was defined as OV/BV > 2%[2]. Means ± standard deviations are displayed. Differences between groups were tested using one-way ANOVA or Kruskal-Wallis *H* test. Exact *p*-values with corresponding effect sizes η^2 (0.02 ≙ small, 0.13 ≙ medium, 0.26 ≙ large effect size) are presented. For sex and osteomalacia absolute values with corresponding percentages are displayed. Differences in the frequency distribution between groups were tested using two-tailed Fisher's exact test. *p*-values with corresponding effect sizes ω (0.1 ≙ small, 0.3 ≙ medium, 0.5 ≙ large effect size) are presented. Numbers in bold indicate statistical significance ($p < 0.05$. Source data are provided as a Source Data file.

Supplementary Table 8. Laboratory bone metabolism in myelofibrosis patients prior to allo-HSCT and at specific time points (30 days, 100 days, and 1 year) subsequent to allo-HSCT.

	**Reference range**	**Pre-HSCT**			**30 days**			**100 days**			**1 year**				
Parameter		Mean	SD	n	Mean	SD	n	Mean	SD	n	Mean	SD	n	*p*	η^2
**Laboratory**															
Calcium (mmol/l)	2.08 - 2.65	2.16	0.17	123	2.19	0.16	120	2.27	0.14	112	2.30	0.15	94	**<0.0001**	0.13
Phosphate (mmol/l)	0.77 - 1.65	1.19	0.22	113	1.21	0.24	49	1.21	0.37	28	1.01	0.28	24	**0.010**	0.04
25-OH-D (µg/l)	> 30	17.9	10.1	96	18.8	8.2	59	22.5	10.3	63	32.0	10.1	77	**<0.0001**	0.24
PTH (ng/l)	17 - 84	109.6	52.2	43	73.5	40.3	29	60.1	40.5	37	44.4	17.1	24	**<0.0001**	0.28
ALP (U/l)	46 - 116	94.9	50.1	123	110.4	68.7	119	118.6	98.8	111	163.2	113.6	92	**<0.0001**	0.09
Bone ALP (U/l)	5.2 - 24.4	13.1	6.7	39	14.3	8.8	29	15.2	8.0	33	29.1	25.5	24	**0.003**	0.09
Osteocalcin (µg/l)	5.4 - 59.1	24.0	14.6	44	34.9	24.2	29	36.6	19.6	33	28.6	11.6	23	**0.005**	0.08
DPD (nmol/mmol)	2 - 7	8.2	5.7	35	12.9	5.3	22	16.1	8.0	25	10.6	5.3	18	**<0.0001**	0.22

Abbreviations: Allo-HSCT, Allogeneic hematopoietic stem cell transplantation. ALP, Alkaline phosphatase. Bone ALP, Bone-specific alkaline phosphatase. DPD, Deoxypyridinoline per creatinine. PTH, Parathyroid hormone. SD, Standard deviation. 25-OH-D, 25-hydroxyvitamin D.

Means ± standard deviations with corresponding sample sizes are displayed. Differences between groups were tested using one-way ANOVA or Kruskal-Wallis *H* test. Exact *p*-values with corresponding effect sizes η^2 (0.02 ≙ small, 0.13 ≙ medium, 0.26 ≙ large effect size) are presented. Numbers in bold indicate statistical significance ($p < 0.05$). Source data are provided as a Source Data file.

Supplementary References

1. Hansen, S., Shanbhogue, V., Folkestad, L., Nielsen, M.M. & Brixen, K. Bone microarchitecture and estimated strength in 499 adult Danish women and men: a cross-sectional, population-based high-resolution peripheral quantitative computed tomographic study on peak bone structure. *Calcif Tissue Int.* **94**, 269-281 (2014).
2. Priemel, M.*, et al.* Bone mineralization defects and vitamin D deficiency: histomorphometric analysis of iliac crest bone biopsies and circulating 25-hydroxyvitamin D in 675 patients. *J Bone Miner Res.* **25**, 305-312 (2010).
