# Supplementary material for: Reconstructing skeletal homeostasis through allogeneic hematopoietic stem cell transplantation in myelofibrosis
Source: Nat Commun. 2025 Jan 20;16:741. doi: 10.1038/s41467-025-55915-w (PMC11747566; doi:10.1038/s41467-025-55915-w)
Supplement: Supplementary file 2 — Reporting Summary [file 41467_2025_55915_MOESM2_ESM.pdf]

nature portfolio

Corresponding author(s): Tim Rolvien, MD, PhD
Nicolaus Kröger, MD

Last updated by author(s): Jan 2, 2025

Reporting Summary

Nature Portfolio wishes to improve the reproducibility of the work that we publish. This form provides structure for consistency and transparency in reporting. For further information on Nature Portfolio policies, see our Editorial Policies and the Editorial Policy Checklist.

Statistics

For all statistical analyses, confirm that the following items are present in the figure legend, table legend, main text, or Methods section.

n/a	Confirmed	
☐	☒	The exact sample size (*n*) for each experimental group/condition, given as a discrete number and unit of measurement
☐	☒	A statement on whether measurements were taken from distinct samples or whether the same sample was measured repeatedly
☐	☒	The statistical test(s) used AND whether they are one- or two-sided<br>*Only common tests should be described solely by name; describe more complex techniques in the Methods section.*
☒	☐	A description of all covariates tested
☐	☒	A description of any assumptions or corrections, such as tests of normality and adjustment for multiple comparisons
☐	☒	A full description of the statistical parameters including central tendency (e.g. means) or other basic estimates (e.g. regression coefficient) AND variation (e.g. standard deviation) or associated estimates of uncertainty (e.g. confidence intervals)
☐	☒	For null hypothesis testing, the test statistic (e.g. F, t, r) with confidence intervals, effect sizes, degrees of freedom and P value noted<br>*Give P values as exact values whenever suitable.*
☒	☐	For Bayesian analysis, information on the choice of priors and Markov chain Monte Carlo settings
☒	☐	For hierarchical and complex designs, identification of the appropriate level for tests and full reporting of outcomes
☐	☒	Estimates of effect sizes (e.g. Cohen's d, Pearson's r), indicating how they were calculated

Our web collection on statistics for biologists contains articles on many of the points above.

Software and code

Policy information about availability of computer code

Data collection	No software was used for data collection.
Data analysis	Data analysis was performed using SPSS Statistics 29.0.2 (IBM, Armonk, NY, USA) and GraphPad Prism 10.4.1 (GraphPad Software, San Diego, CA, USA).

For manuscripts utilizing custom algorithms or software that are central to the research but not yet described in published literature, software must be made available to editors and reviewers. We strongly encourage code deposition in a community repository (e.g. GitHub). See the Nature Portfolio guidelines for submitting code & software for further information.

Data

Policy information about availability of data

All manuscripts must include a data availability statement. This statement should provide the following information, where applicable:

- Accession codes, unique identifiers, or web links for publicly available datasets
- A description of any restrictions on data availability
- For clinical datasets or third party data, please ensure that the statement adheres to our policy

All data supporting the results of this study are available in the article, Supplementary Information and/or Source Data file. Source data are provided with this paper.

Research involving human participants, their data, or biological material

Policy information about studies with human participants or human data. See also policy information about sex, gender (identity/presentation), and sexual orientation and race, ethnicity and racism.

Reporting on sex and gender	Self-reported information was used for the primary categorisation of sex. In addition, patients were karyotyped at the time of diagnosis and gonosomes were available to confirm biological sex (female/male). Groups were matched by propensity score matching for sex, age, and BMI. Further sex-specific data is presented in the supplementary data.
Reporting on race, ethnicity, or other socially relevant groupings	N/A.
Population characteristics	Patients with advanced stage myeloproliferative neoplasms (primary myelofibrosis, post-ET myelofibrosis, post-PV myelofibrosis) undergoing allogeneic hematopoietic stem cell transplantation were recruited from the Department of Stem Cell Transplantation, University Medical Center Hamburg-Eppendorf. Detailed characteristics of the study cohort are presented in the supplementary tables.
Recruitment	See above.
Ethics oversight	Ethikkommission der Ärztekammer Hamburg (2021-100659-BO-ff).

Note that full information on the approval of the study protocol must also be provided in the manuscript.

Field-specific reporting

Please select the one below that is the best fit for your research. If you are not sure, read the appropriate sections before making your selection.

☒ Life sciences ☐ Behavioural & social sciences ☐ Ecological, evolutionary & environmental sciences

For a reference copy of the document with all sections, see nature.com/documents/nr-reporting-summary-flat.pdf

Life sciences study design

All studies must disclose on these points even when the disclosure is negative.

Sample size	All available data were used to maximize the power.
Data exclusions	Patients suffering from disease relapse, secondary acute leukemia, graft failure, or death after transplantation were excluded.
Replication	N/A
Randomization	N/A
Blinding	N/A

Reporting for specific materials, systems and methods

We require information from authors about some types of materials, experimental systems and methods used in many studies. Here, indicate whether each material, system or method listed is relevant to your study. If you are not sure if a list item applies to your research, read the appropriate section before selecting a response.

Materials & experimental systems

n/a	Involved in the study	
☒	☐	Antibodies
☒	☐	Eukaryotic cell lines
☒	☐	Palaeontology and archaeology
☒	☐	Animals and other organisms
☐	☒	Clinical data
☒	☐	Dual use research of concern
☒	☐	Plants

Methods

n/a	Involved in the study	
☒	☐	ChIP-seq
☒	☐	Flow cytometry
☒	☐	MRI-based neuroimaging

Clinical data

Policy information about clinical studies

All manuscripts should comply with the ICMJE guidelines for publication of clinical research and a completed CONSORT checklist must be included with all submissions.

Clinical trial registration	N/A
Study protocol	N/A
Data collection	Patients were recruited for this prospective cohort study between May 2021 to August 2023
Outcomes	Primary: areal bone mineral density (DXA hip and spine), bone volume to tissue volume (histology), fibrosis area to marrow area (histology); Secondary: bone microarchitecture (XCT radius and tibia), matrix mineralization (qBEI), laboratory bone turnover

Plants

Seed stocks	N/A
Novel plant genotypes	N/A
Authentication	N/A
